# Case report: Sequential treatment strategy for advanced basal cell carcinoma in Gorlin-Goltz syndrome: integration of vismodegib, radiotherapy, surgery, and high-intensity focused ultrasound

**DOI:** 10.3389/fonc.2024.1428702

**Published:** 2024-07-18

**Authors:** Jacek Calik, Małgorzata Oślizło, Beata Słocka-Romaniuk, Ahmed Elsaftawy, Natalia Sauer

**Affiliations:** ^1^ Department of Clinical Oncology, Wroclaw Medical University, Wrocław, Poland; ^2^ Old Town Clinic, Wrocław, Poland; ^3^ Faculty of Medicine, Wroclaw Medical University, Wrocław, Poland; ^4^ Department of Radiotherapy, Lower Silesian Center of Pulmonology, Oncology and Hematology in Wrocław, Wrocław, Poland; ^5^ Department of Plastic and Hand Surgery, St. Jadwiga Śląska Hospital, Trzebnica, Poland; ^6^ Department of Clinical Pharmacology, Faculty of Pharmacy, Wroclaw Medical University, Wrocław, Poland

**Keywords:** basal cell carcinoma (BCC), high-intensity focused ultrasound (HIFU), Gorlin-Goltz syndrome, skin cancer, HIFU

## Abstract

Managing advanced basal cell carcinoma (BCC) in patients with Gorlin-Goltz syndrome presents unique clinical challenges due to the tumor’s aggressive nature and potential for widespread metastasis. This case study details a sequential treatment regimen for a 68-year-old female patient with an extensive, inoperable BCC. Employing a multimodal approach that integrates radiotherapy, the Hedgehog pathway inhibitor vismodegib, and High-Intensity Focused Ultrasound (HIFU), we demonstrate the potential for nearly complete remission in a patient with advanced BCC. Initial treatment with radiotherapy and vismodegib reduced tumor size significantly, but the largest mass displayed resistance over time, signifying the need for innovative therapies. Subsequent HIFU treatment targeted individual lesions, showcasing a non-invasive method that provided precise treatment while mitigating systemic side effects. The case emphasizes the necessity of continual adaptation in treatment plans to address the development of resistance and underscores the importance of incorporating new technologies and targeted therapies for complex BCC cases. The successful outcome of this integrated strategy suggests a promising direction for future research and highlights the importance of multidisciplinary approaches that tailor treatment to individual patient needs, tumor characteristics, and evolving therapeutic landscapes.

## 1 Introduction

Basal cell carcinoma (BCC) represents the majority of nonmelanoma skin cancers, constituting about 80% of cases (1, 2). In individuals with Gorlin-Goltz syndrome aged above 20 years, the frequency of BCC is 51.4%, whereas it is 71.7% in patients aged above 40 years (3). Although BCCs are generally characterized by their limited metastatic propensity, they can exhibit local invasiveness and potential for dissemination to distant anatomical sites in the absence of timely and appropriate therapeutic intervention (4). The management of basal cell carcinoma (BCC), particularly in its advanced stages, has evolved significantly with the integration of various treatment modalities, including surgical techniques, radiation therapy, targeted molecular therapies, and immunotherapies (5–10). Surgical intervention remains the primary treatment modality, offering histologic confirmation of tumor clearance (11). Standard excision and Mohs micrographic surgery (MMS) are among the surgical techniques employed, with MMS providing an intraoperative complete margin assessment for certain BCC cases (12, 13).

For more challenging instances of BCC, particularly those that are inoperable or exhibit resistance to conventional treatments, the advent of molecular-targeted therapies has markedly improved prognosis (14). Among these, inhibitors of the Hedgehog signaling pathway, such as vismodegib and sonidegib, have emerged as pivotal in the management of advanced BCCs (15–17). These agents specifically target the aberrant activation of the Hedgehog pathway, a common molecular aberration in BCC, offering a therapeutic option for lesions that are not amenable to surgery or radiotherapy (18). Recent studies have shown that vismodegib has demonstrated efficacy and durability of response in treating advanced BCC, with long-term updates showing sustained effectiveness. Investigator-assessed objective response rates were 48.5% for metastatic BCC (mBCC) and 60.3% for locally advanced BCC (laBCC), with median durations of response being 14.8 months for mBCC and 26.2 months for laBCC (19). The durability of response and long-term safety reinforce vismodegib’s clinical utility in advanced BCC cases where treatment options are limited. However, one of the challenges in the long-term use of vismodegib is the development of resistance, either through acquired mutations in the Smoothened (SMO) gene or the selection of pre-existing SMO mutations within the tumor (20). Secondary resistance is estimated to occur in 15-35% of patients who receive vismodegib as a retreatment after initial discontinuation due to relapse. Some mutations, such as D473H, can confer resistance to both vismodegib and sonidegib.

In this context, we present a clinical case study of a patient with a giant BCC that was treated over more than two years. This case exemplifies how the integration of radiotherapy and Hedgehog pathway inhibitor treatment, followed by the innovative application of local High-Intensity Focused Ultrasound (HIFU) therapy, can lead to nearly complete recovery of the patient. It highlights the importance of a synergistic approach that combines different treatment techniques to achieve a favorable outcome.

## 2 Case description

**Figure 1:**
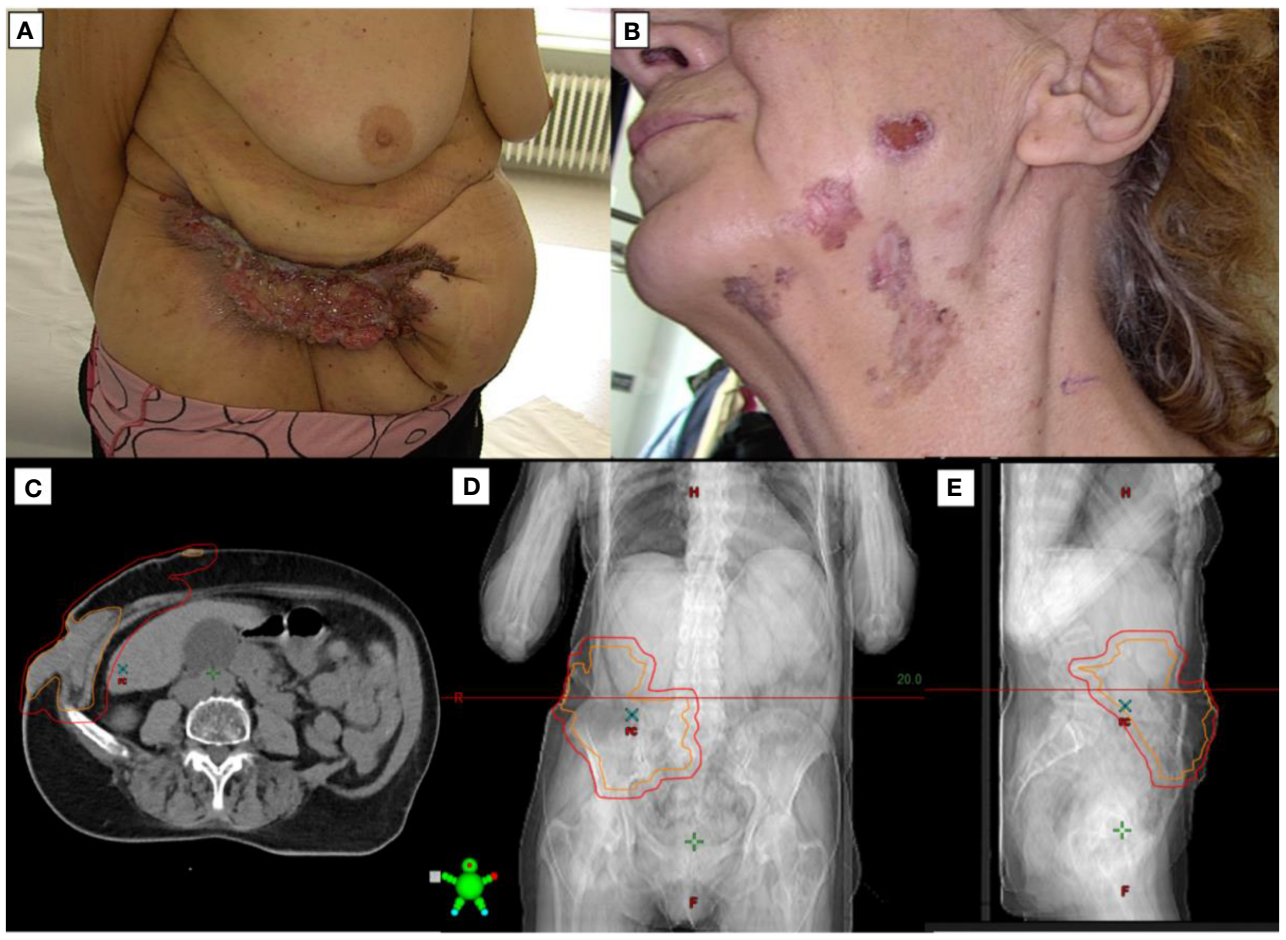
Overview of a patient with giant BCC before treatment. **(A)** An 68-year-old woman presented with an enormous, fetid, abdominal wall mass. **(B)** Lesions of BCC on the face and neck. **(C)** Axial CT of the tumor, **(D)** Coronal CT of the tumor, **(E)** Sagittal CT of the tumor.

A 68-year-old female patient of Caucasian ethnicity presented with advanced, multifocal cutaneous lesions encompassing the entire body, including facial involvement (**Figures 1A, B**). The primary lesions were initially located in the abdominal area, face, neck and back. A biopsy of the mass was performed on 16 January 2022, and histopathological examination revealed an infiltrative basal cell carcinoma. The computed tomography performed on 6 April 2022 revealed an infiltrative mass within the intra-abdominal and pelvic regions, measuring 11.4 x 3.1 x 12 cm, which infiltrates the skin and muscle layers (**Figures 1C–E**).This patient was deemed inoperable due to the extensive nature of the disease. Additionally, the patient was diagnosed with Gorlin-Goltz syndrome, a rare genetic disorder characterized by a predisposition to multiple neoplastic and non-neoplastic tumors, further complicating her clinical presentation due to the disease’s multifocality and the presence of a large, partially necrotic tumor exuding a malodorous discharge.

In response to the advanced stage of the tumor and its symptomatic impact, the patient underwent conformal radiotherapy employing 6MeV photons, targeting the neoplastic infiltration of the lower abdominal skin with a calculated total dose of 20 Gy delivered in 5 fractions from March 28 to April 1, 2022. Following radiotherapy on April 19, 2022, treatment with vismodegib, a Hedgehog pathway inhibitor approved for inoperable metastatic basal cell carcinoma, was initiated at a dose of 150 mg per day. The treatment was well-tolerated, apart from intermittent episodes of nausea. Simultaneously, the patient underwent treatment for iron deficiency anemia by orally administering a liquid formulation containing 40mg of iron protein succinate per 15ml, aiming to enhance overall health and facilitate recovery throughout cancer therapy.

**Figure 2:**
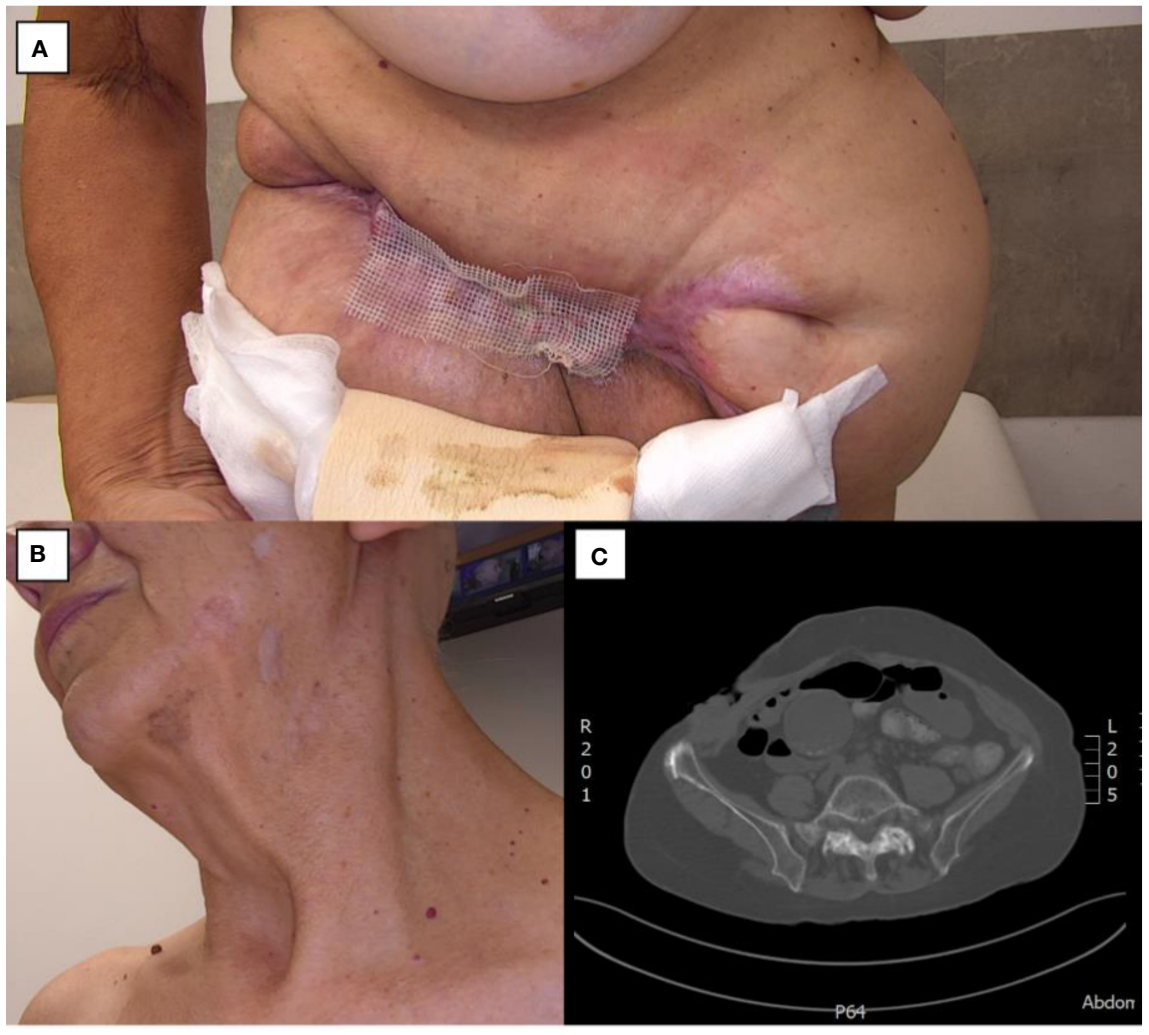
Giant BCC during treatment. **(A)** Abdominal wall mass. **(B)** Regressive lesions on the face and neck. **(C)** Axial CT scan of the abdominal mass.

The treatment with vismodegib was extended until December 2023, yielding a partial regression of the tumor burden. However, as no further improvement was observed, the therapy was discontinued in December 7th. Remarkably, certain flat lesions underwent near-complete resolution (**Figure 2B**). A subsequent computed tomography scan (December 19, 2023) revealed the presence of an infiltrative mass measuring 10.8x2.2x11 cm within the abdomen and pelvis (**Figure 2C**).

However, the largest tumor, after initially responding with a reduction in size demonstrated progressive growth from July 11, 2023. Given the documented transient efficacy of vismodegib, typically lasting less than 12 months, and considering the patient’s prolonged treatment duration exceeding one year, a multidisciplinary consultation was convened to evaluate surgical excision of the largest tumor as a potential therapeutic strategy (**Figure 2A**). The aim was to facilitate subsequent management of residual lesions through adjuvant modalities such as photodynamic therapy, high-frequency ultrasound, or topical fluorouracil (Efudix).

Subsequent to consultations across multiple specialized centers, a decision for surgical intervention was made by a facility proficient in plastic surgery located in Trzebnica. The surgery was performed on December 27. The procedure involved excising a segment of the tumor-invaded bone, abdominal wall, musculature, omentum, and regional lymph nodes. The surgical outcome was classified as an R0 resection, indicating the removal of the tumor with clear margins within healthy tissue boundaries. Post-operative recovery was challenged by significant tissue tension and delayed wound healing, necessitating reoperation and vacuum-assisted closure therapy, which subsequently led to a transient decline in the patient’s clinical status. Nevertheless, comprehensive rehabilitation efforts facilitated a gradual return to baseline function.

**Figure 3:**
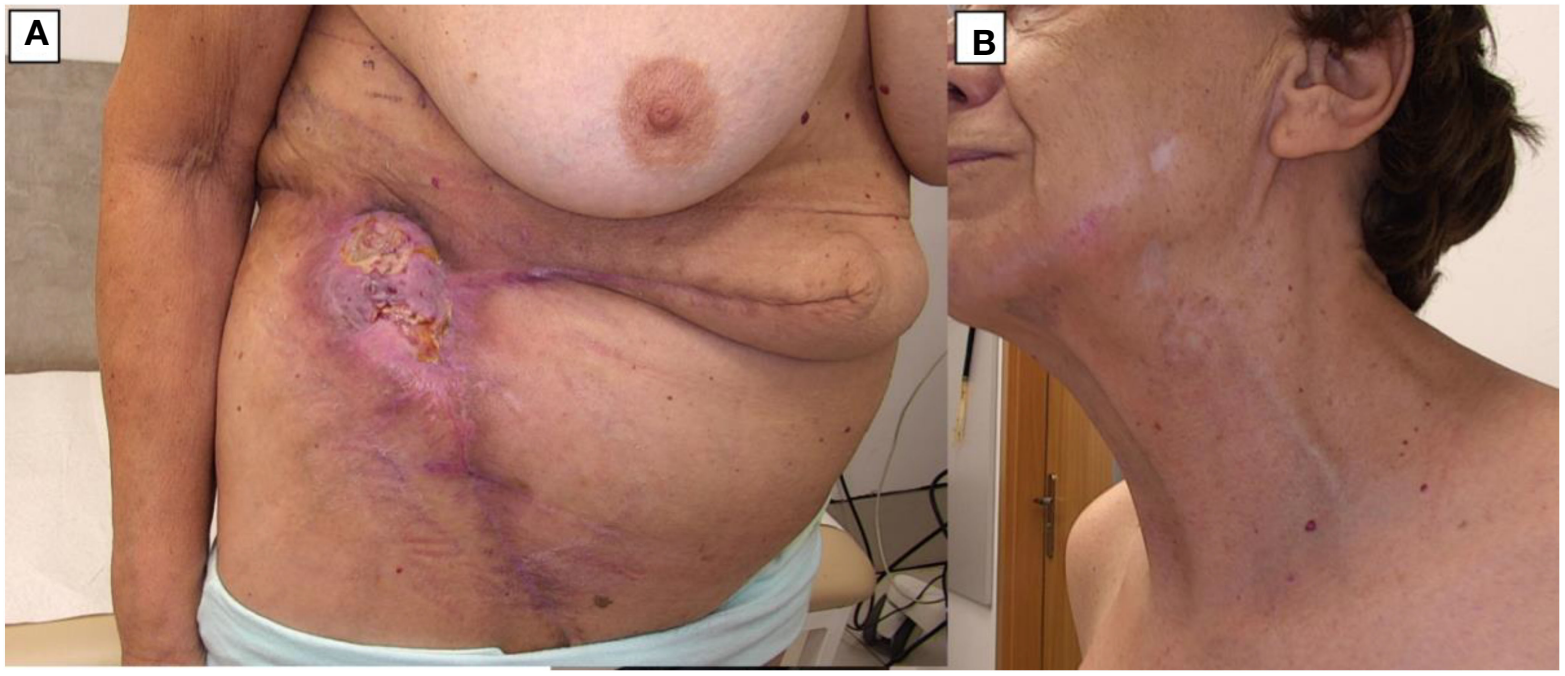
BCC during treatment (March 2024). **(A)** Lesion on the abdomen after skin grafting. **(B)** The skin of the face and neck with significant regression of most lesions.

Skin reconstruction at the site of the largest tumor was performed using split-thickness skin grafts on February 2 and February 26, 2024 (**Figure 3A**).

Dermatoscopic evaluation post-treatment revealed that lesions previously identified as basal cell carcinoma, exhibiting characteristic dermatoscopic features, had achieved complete remission and were fully cicatrized (**Figure 3B**). Conversely, lesions persisting with dermatoscopic indications of active neoplastic infiltration underwent ultrasound assessment using the Dermascan^®^C system (Cortex Technology ApS, Aalborg, Denmark) revealing infiltrative depths ranging from 0.4 mm to 0.8 mm, thereby classifying these as superficial basal cell carcinoma.

**Figure 4:**
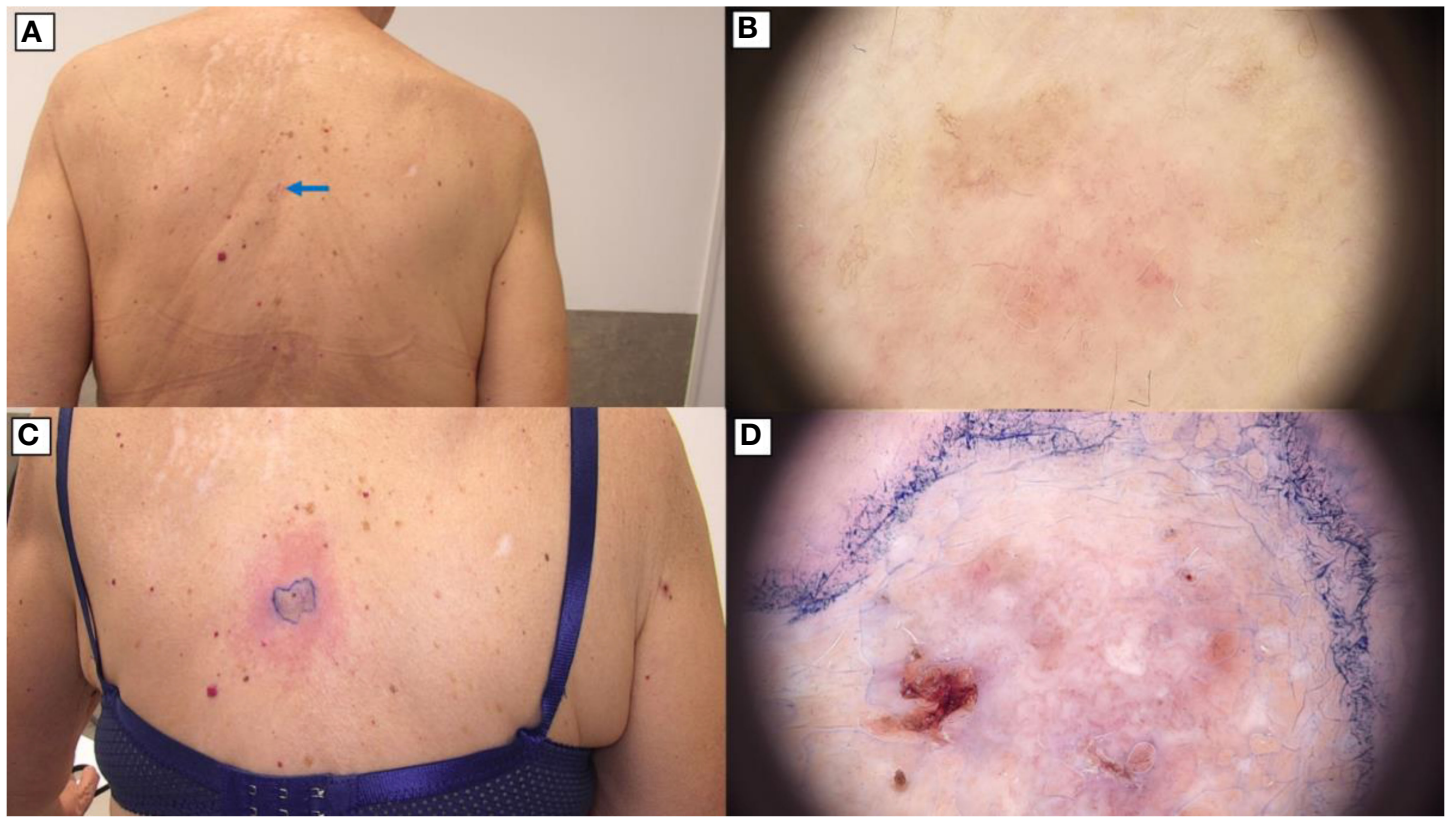
BCC on the back treated with High-Intensity Focused Ultrasound (HIFU). **(A)** Macroscopic view before HIFU treatment.; **(B)** Dermoscopic view before HIFU treatment.; **(C)** Macroscopic view immediately after HIFU treatment.; **(D)** Dermoscopic view immediately after HIFU treatment.

**Figure 5:**
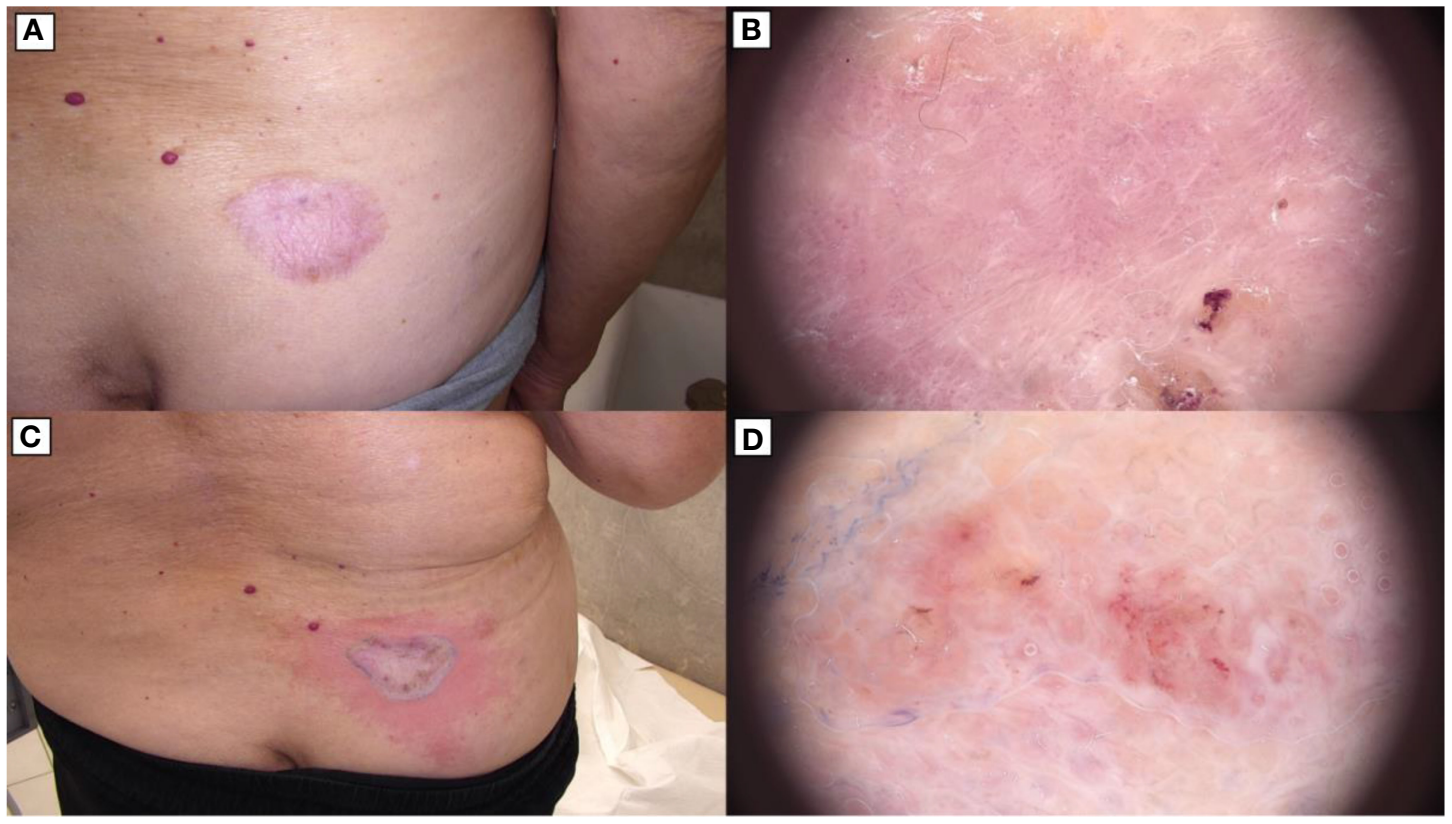
BCC on the back treated with High-Intensity Focused Ultrasound (HIFU). **(A)** Macroscopic view before HIFU treatment.; **(B)** Dermoscopic view of the back before HIFU treatment.; **(C)** Macroscopic view immediately after HIFU treatment.; **(D)** Dermoscopic view of the back immediately after HIFU treatment.

The patient was subsequently enrolled in a clinical trial (NCT05698706) aimed at addressing individual basal cell carcinoma (BCC) lesions located on the lower and upper back which had not regressed after prior therapy with vismodegib(**Figures 4**, **5**). The ONE-M system (TOOsonix A/S, Hoersholm, Denmark), operating at a frequency of 20 MHz, was utilized for High-Intensity Focused Ultrasound (HIFU) therapy. Treatment parameters for HIFU were individually tailored to the specific characteristics of each cutaneous lesion. The energy delivered by HIFU ranged from 0.7 to 1.3 J per exposure, with a duration of 150 ms for each dose. Treatments were carried out in a contiguous fashion, ensuring a 1 to 2 mm separation between doses for comprehensive lesion coverage, while maintaining a minimal circumferential margin of approximately 1 mm. The interval between subsequent exposures was maintained at around 1-2 seconds.

The management of giant basal cell carcinoma (BCC) poses significant challenges due to the tumor’s extensive size, potential for local invasion, and the risk of metastasis (21, 22). In the case presented, a multimodal treatment approach integrating radiotherapy, Vismodegib, and High-Intensity Focused Ultrasound (HIFU) was employed, reflecting a tailored strategy aimed at addressing the complexities associated with advanced BCC. This approach is supported by the examination of various case reports highlighting the necessity for innovative and individualized treatment modalities in managing giant BCC (23–27).

Vismodegib has shown significant efficacy in treating locally advanced BCC that is not amenable to surgery or radiation, as well as in cases of metastatic BCC [25], [28]. It has been proven to reduce tumor size and inhibit further tumor growth. The use of vismodegib as a neoadjuvant treatment can decrease the size of the tumor, potentially allowing for less extensive surgery and better cosmetic outcomes (26).

However, the journey with vismodegib is not devoid of obstacles. Among the challenges are adverse effects such as muscle spasms, hair loss, changes in taste, weight loss, and fatigue, which can significantly detract from a patient’s quality of life and sometimes necessitate the discontinuation of treatment (28). Resistance to vismodegib can also develop over time, complicating treatment efficacy and highlighting the necessity for continuous research to understand and counteract these resistance mechanisms (29). Additionally, the cost and availability of vismodegib pose considerable barriers, especially in areas with limited healthcare resources or where the drug has not been approved, making accessibility a significant concern (30). Thus, the incorporation of adjunctive therapies, including radiotherapy and molecular-targeted agents, plays a critical role in enhancing treatment outcomes and minimizing the likelihood of recurrence (31).

High-Intensity Focused Ultrasound (HIFU), as applied in our case, represents an innovative approach to the local management of BCC lesions, offering a non-invasive option for tumors that are challenging to treat surgically (32). High-Intensity Focused Ultrasound (HIFU) employs focused ultrasound waves generated by a transducer to precisely target a specific focal point within the tissue (33–35). This concentrated energy induces two principal effects: thermal and mechanical. The thermal effect is characterized by a rapid increase in temperature at the focal point, resulting in coagulative necrosis and irreversible cellular destruction. The mechanical effect involves cavitation, wherein the formation of microbubbles within the tissue leads to cellular disruption. The HIFU system functions by converging ultrasound waves to create a high-intensity focal zone. At the focal point, the energy density is significantly amplified, causing localized heating and mechanical stress that culminates in tissue ablation. This process is analogous to focusing light through a magnifying glass, but instead of light, it utilizes ultrasound waves. HIFU systems can be calibrated to elicit varying degrees of thermal and mechanical effects by adjusting the power and pulse duration settings. This capability allows for a spectrum of responses, ranging from mild localized heating to comprehensive tissue ablation. Furthermore, the non-invasive nature of HIFU offers a substantial advantage in treating various conditions without necessitating surgical intervention, thereby reducing potential side effects and complications commonly associated with invasive procedures (34, 36).

This method allows for the precise treatment of individual lesions that were not previously cured by vismodegib, while simultaneously sparing the patient from the systemic effects of the drug and avoiding numerous adverse side effects.

This method allows for precise treatment of individual lesions that were not previously cured by vismodegib, while simultaneously sparing the patient from the systemic effects of the medication and avoiding numerous side effects.

Importantly, this aligns with emerging evidence suggesting the benefit of incorporating advanced technologies and targeted therapies in the treatment of complex BCC cases (32). Our previous experience has demonstrated that HIFU exhibits a high success rate in completely ablating BCC lesions, with no recurrences observed during the six-month follow-up period (37). HIFU treatment is associated with minimal adverse events and superior cosmetic outcomes, significantly enhancing patient satisfaction compared to conventional treatments. Therefore, our results suggest that integrating HIFU into the therapeutic arsenal against BCC offers a non-invasive, patient-preferred treatment modality that provides a promising balance between clinical efficacy, safety, and aesthetic preservation. Recent studies have indicated that HIFU possesses immunomodulatory capabilities, although this aspect is currently undergoing in-depth investigation (38).

The discussion of these cases within the scientific community serves not only to share insights into the management of giant BCC but also to stimulate further research into optimal treatment combinations and strategies. Future studies should aim to refine these approaches, exploring the potential for integrating new molecular-targeted therapies, enhancing surgical techniques, and evaluating the long-term outcomes of non-invasive treatments like HIFU. Additionally, the development of multidisciplinary treatment protocols that consider the tumor’s characteristics, patient preferences, and potential impact on quality of life will be crucial in advancing the care of individuals with giant BCC. In conclusion, the management of giant basal cell carcinoma requires a comprehensive, tailored approach that leverages the strengths of surgical intervention, targeted molecular therapies, and innovative technologies like HIFU. At the time of qualification for treatment, only one line of treatment with Vismodegib was available in Poland, sonidegib was not yet available in Poland, precluding its use in this case. The second line of treatment with cemiplimab or pembrolizumab was not initiated because the tumor mass reduction enabled local treatment, which is the treatment of choice, provided it aims for radical treatment. The inclusion of HIFU provided a significant benefit by avoiding the systemic effects associated with immunotherapy. This approach allowed for the precise, localized treatment of lesions, effectively curing them without exposing the patient to the potential adverse systemic effects commonly seen with immunotherapy. This ensured both efficacy and minimized the overall burden on the patient, demonstrating the potential of HIFU as a valuable tool in the management of BCC. By adapting treatment strategies to the complexities of each case and remaining vigilant for the emergence of resistance or metastasis, it is possible to achieve favorable outcomes for patients facing this challenging condition.

## 3 Conclusions

The comprehensive management of giant basal cell carcinoma (BCC) necessitates a multimodal treatment approach that effectively leverages the synergistic potentials of radiotherapy, targeted molecular therapies such as Vismodegib, and innovative non-invasive treatments like High-Intensity Focused Ultrasound (HIFU). This case study illustrates a successful application of such an integrated treatment strategy, emphasizing the importance of addressing the complexities associated with advanced BCC to achieve favorable outcomes.

## Data availability statement

The raw data supporting the conclusions of this article will be made available by the authors, without undue reservation.

## Ethics statement

Ethical approval was not required for the studies involving humans because it is a case report with retrospective data of standard treatment. The studies were conducted in accordance with the local legislation and institutional requirements. The participants provided their written informed consent to participate in this study. Written informed consent was obtained from the individual(s) for the publication of any potentially identifiable images or data included in this article.

## Author contributions

JC: Conceptualization, Formal analysis, Investigation, Methodology, Project administration, Resources, Supervision, Validation, Writing – original draft, Writing – review & editing. MO: Conceptualization, Validation, Visualization, Writing – original draft, Writing – review & editing. BS-R: Resources, Writing – original draft, Writing – review & editing. NS: Conceptualization, Data curation, Formal analysis, Funding acquisition, Investigation, Methodology, Project administration, Resources, Software, Supervision, Validation, Visualization, Writing – original draft, Writing – review & editing. AE: Writing – review & editing, Supervision, Formal analysis, Project administration, Investigation, Funding acquisition.

## References

[1] Piva De Freitas P Senna CG Tabai M Chone CT Altemani A. Metastatic basal cell carcinoma: A rare manifestation of a common disease. Case Rep Med. (2017) 2017:8929745. doi: 10.1155/2017/8929745 29279714 PMC5723960

[2] Khayyati Kohnehshahri M Sarkesh A Mohamed Khosroshahi L HajiEsmailPoor Z Aghebati-Maleki A Yousefi M. Current status of skin cancers with a focus on immunology and immunotherapy. Cancer Cell Int. (2023) 23:1–15. doi: 10.1186/s12935-023-03012-7 37605149 PMC10440946

[3] Şereflican B Tuman B Şereflican M Halıcıoğlu S Özyalvaçlı G Bayrak S. Gorlin-goltz syndrome. Turkish Arch Pediatr Pediatr Arşivi. (2017) 52:173. doi: 10.5152/TurkPediatriArs. PMC5644586 29062253

[4] Qadir MI. Skin cancer: Etiology and management. Pak J Pharm Sci. (2016) 29:999–1003. 27166545

[5] Cho M Gordon L Rembielak A Woo TCS. Utility of radiotherapy for treatment of basal cell carcinoma: a review. Br J Dermatol. (2014) 171:968–73. doi: 10.1111/bjd.13253 25041560

[6] Luz FB Cardoso GP Ferron C. Surgical treatment of basal cell carcinoma: an algorithm based on theliterature. Bras Dermatol. (2015) 90:377. doi: 10.1590/abd1806-4841.20153304 PMC4516103 26131869

[7] Koelblinger P Lang R. New developments in the treatment of basal cell carcinoma: Update on current and emerging treatment options with a focus on vismodegib. Onco Targets Ther. (2018) 11:8327–40. doi: 10.2147/OTT PMC6267762 30568456

[8] Hoashi T Kanda N Saeki H. Molecular mechanisms and targeted therapies of advanced basal cell carcinoma. Int J Mol Sci. (2022) 23:11968. doi: 10.3390/ijms231911968 36233269 PMC9570397

[9] Belzer A Pach J Mortlock RD Clune J Olino K Sznol M. Evaluating the medical management of locally advanced and metastatic basal cell carcinoma: A single institutional retrospective analysis investigating efficacy, safety, and tolerability. JAAD Int. (2023) 11:174. doi: 10.1016/j.jdin.2023.02.007 37252181 PMC10213716

[10] Dessinioti C Stratigos AJ. Immunotherapy and its timing in advanced basal cell carcinoma treatment. Dermatol Pract Concept. (2023) 13:e2023252-e2023252. doi: 10.5826/dpc.1304a252 37992360 PMC10656142

[11] Chen OM Kim K Steele C Wilmas KM Aboul-Fettouh N Burns C. Advances in management and therapeutics of cutaneous basal cell carcinoma. Cancers (Basel). (2022) 14:3720. doi: 10.3390/cancers14153720 35954384 PMC9367462

[12] Van Loo E Mosterd K Krekels GAM Roozeboom MH Ostertag JU Dirksen CD. Surgical excision versus Mohs’ micrographic surgery for basal cell carcinoma of the face: A randomised clinical trial with 10 year follow-up. Eur J Cancer. (2014) 50:3011–20. doi: 10.1016/j.ejca.2014.08.018 25262378

[13] Alsaif A Hayre A Karam M Rahman S Abdul Z Matteucci P. Mohs micrographic surgery versus standard excision for basal cell carcinoma in the head and neck: systematic review and meta-analysis. Cureus. (2021) 13. doi: 10.7759/cureus.19981 PMC8715344 34984139

[14] Cowey CL. Targeted therapy for advanced basal-cell carcinoma: vismodegib and beyond. Dermatol Ther (Heidelb). (2013) 3:17. doi: 10.1007/s13555-013-0019-9 23888252 PMC3680638

[15] Dummer R Ascierto PA Basset-Seguin N Dréno B Garbe C Gutzmer R. Sonidegib and vismodegib in the treatment of patients with locally advanced basal cell carcinoma: a joint expert opinion. J Eur Acad Dermatol Venereol. (2020) 34:1944–56. doi: 10.1111/jdv.16230 31990414

[16] García Ruiz AJ García-Agua Soler N Herrera Acosta E Zalaudek I Malvehy J. Benefit–risk assessment of sonidegib and vismodegib in the treatment of locally advanced basal cell carcinoma. Drugs Context. (2022) 11. doi: 10.7573/dic.2022-1-2 PMC9281973 35912002

[17] Nazzaro G Benzecry V Mattioli MA Denaro N Beltramini GA Marzano AV. Sonidegib in locally advanced basal cell carcinoma: A monocentric retrospective experience and a review of published real-life data. Cancers (Basel). (2023) 15:3621. doi: 10.3390/cancers15143621 37509282 PMC10377077

[18] Doan HQ Chen L Nawas Z Lee HH Silapunt S Migden M. Switching Hedgehog inhibitors and other strategies to address resistance when treating advanced basal cell carcinoma. Oncotarget. (2021) 12:2089. doi: 10.18632/oncotarget.v12i20 34611482 PMC8487719

[19] Sekulic A Migden MR Basset-Seguin N Garbe C Gesierich A Lao CD. Long-term safety and efficacy of vismodegib in patients with advanced basal cell carcinoma: Final update of the pivotal ERIVANCE BCC study. BMC Cancer. (2017) 17:1–10. doi: 10.1186/s12885-017-3286-5 28511673 PMC5433030

[20] Bossi P Ascierto PA Basset-Seguin N Dreno B Dummer R Hauschild A. Long-term strategies for management of advanced basal cell carcinoma with hedgehog inhibitors. Crit Rev Oncol Hematol. (2023) 189:104066. doi: 10.1016/j.critrevonc.2023.104066 37442495

[21] Lear JT. Challenges and new horizons in the management of advanced basal cell carcinoma: a UK perspective. Br J Cancer. (2014) 111 (8):1476. doi: 10.1038/BJC.2014.270 25211660 PMC4200081

[22] Szewczyk M. Local recurrence risk in head and neck basal cell carcinoma. Pol J Otolaryngol. (2022) 76 (4):30–35. doi: 10.5604/01.3001.0015.8568 36047328

[23] Ceilley RI Del Rosso JQ. Current modalities and new advances in the treatment of basal cell carcinoma. Int J Dermatol. (2006) 45 (5):489–498. doi: 10.1111/J.1365-4632.2006.02673.X 16700779

[24] Warbrick-Smith J O’Neill JK Wilson P. Case Report: Giant anterior chest wall basal cell carcinoma: a reconstructive challenge and review of the literature. BMJ Case Rep. (2013). doi: 10.1136/BCR-2013-008871 PMC3645802 23598936

[25] Chun-Guang M. Successful Treatment of Giant Basal Cell Carcinoma with Topical Imiquimod 5% Cream with Long Term Follow-up. Indian J Dermatol. (2014). 59 (6):575. doi: 10.4103/0019-5154.143520 25484387 PMC4248494

[26] Martins PC. Basal cell carcinoma: multimodal treatment and the role of neoadjuvant vismodegib. Autops Case Rep. (2019). 9 (4). doi: 10.4322/ACR.2019.116 PMC6771444 31641658

[27] Cantisani C. Management of patients with giant basal cell carcinoma during SARS COV2 outbreak in Italy. Transl Biophotonics. (2022). 4 (3). doi: 10.1002/TBIO.202200009 PMC9350373 35942364

[28] Frampton JE Basset-Séguin N. Vismodegib: A Review in Advanced Basal Cell Carcinoma. Drugs. (2018). 78 (11):1145–1156. doi: 10.1007/S40265-018-0948-9 30030732

[29] Sinx KAE. Vismodegib-resistant basal cell carcinomas in basal cell nevus syndrome: Clinical approach and genetic analysis. JAAD Case Rep. (2018). 45 (5):408. doi: 10.1016/J.JDCR.2017.11.011 PMC6031482 29984265

[30] Poggi l Kolesar JM. Vismodegib for the treatment of basal cell skin cancer. Am J Health Syst Pharm. (2013). 70 (12):1033–1038. doi: 10.2146/AJHP120311 23719880

[31] Sahned J. Giant Ulcerative Basal Cell Carcinoma with Local Metastasis: A Case Report and Assessment of Surgical Techniques. Cureus. (2019). 11 (12). doi: 10.7759/CUREUS.6426 PMC6970455 31993264

[32] Serup J Bove T Zawada T Jessen A Poli M. High-frequency (20 MHz) high-intensity focused ultrasound: New Treatment of actinic keratosis, basal cell carcinoma, and Kaposi sarcoma. An open-label exploratory study. Skin Res Technol. (2020) 26:824–31. doi: 10.1111/srt.12883 PMC7754281 32557832

[33] Bove T Zawada T Serup J Jessen A Poli M. High-frequency (20-MHz) high-intensity focused ultrasound (HIFU) system for dermal intervention: Preclinical evaluation in skin equivalents. Skin Res Technol. (2019) 25:217–28. doi: 10.1111/srt.12661 30620418

[34] Calik J Zawada T Bove T Dzięgiel P Pogorzelska-Antkowiak A Mackiewicz J. Healing process after high-intensity focused ultrasound treatment of benign skin lesions: dermoscopic analysis and treatment guidelines. J Clin Med. (2024) 13:931. doi: 10.3390/jcm13040931 38398246 PMC10888560

[35] Bove T Zawada T Serup J Jessen A Poli M. High-frequency (20 MHz) focused ultrasound: A novel method for noninvasive tattoo removal. Curr Probl Dermatol. (2022) 56:268–80. doi: 10.1159/000521487 37263206

[36] Wozniak B Bove T Zawada T Calik J. Treatment of cutaneous neurofibromas in patients with neurofibromatosis type 1. Case Rep Dermatol. (2023) 15:194–201. doi: 10.1159/000534270 37899948 PMC10601743

[37] Calik J Sauer N Woźniak B Wojnar A Pietkiewicz P Dzięgiel P. Pilot study on high-intensity focused ultrasound (HIFU) for basal cell carcinoma: effectiveness and safety. J Clin Med. (2024) 13:3277. doi: 10.3390/jcm13113277 38892988 PMC11173122

[38] Calik J Zawada T Sauer N Bove T. High intensity focused ultrasound (20 MHz) and cryotherapy as therapeutic options for granuloma annulare and other inflammatory skin conditions. Dermatol Ther. (2024) 14:1189–210. doi: 10.1007/s13555-024-01163-7 PMC11116313 38703308

